# Robotic hernia repair III. English version: Robotic incisional hernia repair with transversus abdominis release (r‑TAR). Video report and results of a cohort study

**DOI:** 10.1007/s00104-021-01500-y

**Published:** 2021-09-08

**Authors:** Ulrich A. Dietz, O. Yusef Kudsi, Miguel Garcia-Ureña, Johannes Baur, Michaela Ramser, Sladjana Maksimovic, Nicola Keller, Jörg Dörfer, Lukas Eisner, Armin Wiegering

**Affiliations:** 1 Department of Visceral, Vascular and Thoracic Surgery, Cantonal Hospital Olten (soH), Baslerstrasse 150, 4600 Olten, Switzerland; 2 Department of Surgery, Good Samaritan Medical Center, 235 North Pearl St., 02301 Brockton, MA USA; 3 Hospital Universitario del Henares, Universidade Francisco de Vitoria, 28223 Pozuelo de Alarcón, Madrid Spain; 4 Department of General, Visceral and Vascular Surgery, Cantonal Hospital Baden, Im Engel 1, 5404 Baden, Switzerland; 5 Department of General, Visceral, Transplant, Vascular and Pediatric Surgery, University Hospital Wuerzburg, Oberduerrbacher Strasse 6, 97080 Wuerzburg, Germany

**Keywords:** Robotic surgical procedures, Incisional hernia, Ventral hernia, Retromuscular mesh, Posterior component separation, Robotik, Inzisionale Hernie, Ventrale Hernie, Retromuskuläres Netz, Posteriore Komponentenseparation

## Abstract

The principle of targeted separation or weakening of individual components of the abdominal wall to relieve tension in the median line during major abdominal reconstruction has been known for over 30 years as anterior component separation (aCS) and is an established procedure. In search of alternatives with lower complication rates, posterior component separation (pCS) was developed; transversus abdominis release (TAR) is a nerve-sparing modification of pCS. With the ergonomic resources of robotics (e.g., angled instruments), TAR can be performed in a minimally invasive manner (r-TAR): hernia gaps of up to 14 cm can be closed and a large extraperitoneal mesh implanted. In this video article, the treatment of large incisional hernias using the r‑TAR technique is presented. Exemplary results of a cohort study in 13 consecutive patients are presented. The procedure is challenging, but our own results—as well as reports from the literature—are encouraging. The r‑TAR is becoming the pinnacle procedure for abdominal wall reconstruction.

**Supplementary Information:**

The online version of this article (10.1007/s00104-021-01500-y) includes a video and an intraoperative checklist.

## Abstract (de)

Das Prinzip der gezielten Trennung bzw. Schwächung einzelner Komponenten der Bauchdecke zur Spannungsentlastung der Medianlinie bei großen abdominellen Rekonstruktionen ist seit über 30 Jahren als anteriore Komponentenseparation (aKS) bekannt und ein etabliertes Verfahren. Auf der Suche nach Alternativen mit geringerer Komplikationsrate wurde die posteriore Komponentenseparation (pKS) entwickelt; der „transversus abdominis release“ (TAR) ist eine nervenschonende Modifikation der pKS. Mit den ergonomischen Ressourcen der Robotik (z. B. abgewinkelte Instrumente) kann der TAR minimal-invasiv durchgeführt werden (r-TAR): Bruchlücken von bis zu 14 cm lassen sich verschließen und ein großes extraperitoneales Netz implantieren. In diesem Videobeitrag wird die Versorgung großer Inzisionalhernien in der r‑TAR-Technik präsentiert. Exemplarisch werden die Ergebnisse einer Kohortenstudie an 13 konsekutiven Patienten vorgestellt. Der Eingriff ist anspruchsvoll, die eigenen Ergebnisse sind – wie auch die Berichte aus der Literatur – ermutigend. Der r‑TAR entwickelt sich zur Königsdisziplin der Bauchdeckenrekonstruktion.

## Background

Reconstruction of large incisional hernias remains a challenge despite all the advances of the past decades: the patient’s risk profile, hernia findings, and what is technically feasible converge in the hands of the expert to a treatment plan acceptable to the patient, but there are many gray areas and nuances that also have to be taken into consideration. Although conventional laparoscopic procedures significantly reduce the risk of complications, intraperitoneal mesh placement and a somewhat higher rate of recurrence remain unresolved issues [1].

To revisit the topic of incisional hernia repair would be without greater benefit if the challenges of its surgical therapy were not so current, if innovative therapeutic procedures were not constantly expanding the surgical spectrum, and if new knowledge were not constantly being gained in the field of individual therapy. Robotics—as a highly precise form of laparoscopy—is assuming an increasingly important role in hernia surgery. This video article is the third in a series on robotic hernia surgery and covers robotic transversus abdominis release (r-TAR). Parts I and II discuss inguinal hernia repair (robotic transabdominal preperitoneal patch plasty, r‑TAPP; [2]) and primary ventral and repair of smaller incisional hernias (robotic ventral transabdominal preperitoneal patch plasty [rv-TAPP] and r‑Rives/robotic transabdominal retromuscular umbilical patch plasty [TARUP]; [3]).

## Indications and contraindications

The indications for endoscopic robotic repair of large incisional hernias are in principle similar to those for conventional laparoscopic procedures and also depend on the patient’s risk profile [4, 5]. Incisional hernias with a width of 8–14 cm are a suitable indication for robotic surgery. For smaller hernias, robotic Rives (r-Rives, for incisional hernias) and robotic ventral TAPP (rv-TAPP, for primarily ventral hernias) should be considered as alternatives [3]. The length of the hernia is not as significant in the choice of procedure because the dissection in r‑TAR is comprehensive from subxiphoid to the retropubic space (Retzius’ space).

Relative contraindications are very slim patients, combined median and lateral hernia gaps, and after open abdomen therapy with skin mesh graft coverage of the intestinal convolute (synonym: Thiersch plasty, after Karl Th. Thiersch, 1886).

## Patient information

The minimally invasive procedure and the use of the surgical robot are presented. Regarding the use of the robot, we explain to the patients that it is not an actual robot, but a precision instrument that is guided exclusively by surgeons. General information is given about postoperative complications such as postlaparoscopic shoulder pain, postoperative bleeding, seroma development, and the occurrence of chronic pain or numbness of the skin. In the case of a slim body, there may be a bulge in the area of the skin over the hernia repair, which is very likely to smooth out completely during the first 3–6 months postoperatively.

The Veres needle puncture site on the left subcostal and shaving of the abdomen and right thigh (for the neutral electrode) are addressed. The available results of conventional repairs are mentioned as the expected recurrence rate (approximately 2–8% at 5 years). Implantation of a nonabsorbable, flat, large-pored mesh is discussed.

Patients are advised about options to optimize the cosmetic results of the scars of the skin.

## Anesthesia and positioning

On the day of surgery, in the admission ward, a final consultation is held with the patient, the hernia gap is marked on the skin, and written consent is checked. The abdominal wall is accessed from both sides of the patient; the DaVinci Xi (Intuitive Surgical, CA, USA) is approached from the patient’s feet. The patient is positioned supine on the operating table (Trumpf Medical, Saalfeld, Germany); the way the arms are positioned is not relevant for this procedure. The face and ventilation tube are protected with a metal frame mounted on the operating table. The procedure is performed under general anesthesia; relaxation must be optimal until the end of the procedure or until undocking of the robotic system; if necessary, neuromuscular blockade is antagonized at the end of the procedure. Patients receive perioperative antibiotic prophylaxis with cefuroxime 1.5 g (alternatively clindamycin 600 mg).

## Overview of the relevant anatomy of the transversus abdominis release

**Fig. 1:**
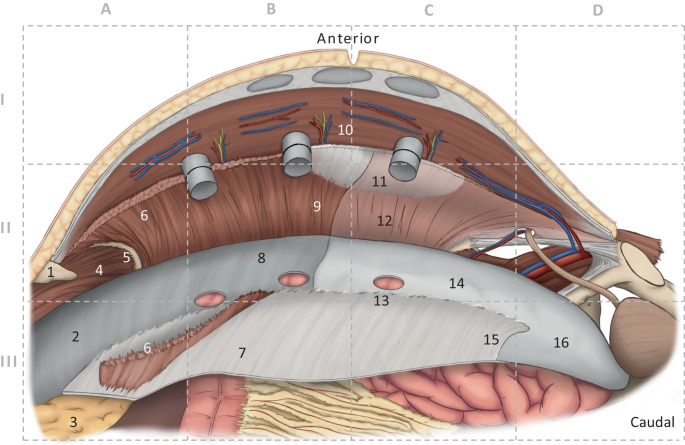
Anatomy of the robotic transversus abdominis release (r-TAR). Intraoperative view of the left side of the abdominal wall; the right (contralateral) side was previously prepared over the three visible trocars (12, 8, and 8 mm). The grid is used as a guide in Figs. 2 and 3. Anatomical structures: *1* xiphoid, *2* fascia diaphragmatica, *3* retroxiphoidal-preperitoneal fat (fatty triangle), *4* diaphragm, 5 costal arch margin, *6* detached transversus abdominis in the area of its insertion onto the posterior rectus sheath, *7* posterior rectus sheath, *8* endoabdominal fascia with peritoneum, *9* transversus abdominis cleared from its fascia (typical preparation layer in the cranial region), *10* intercostal nerves, *11* linea semilunaris, *12* transversus abdominis with its fascia (typical preparation layer in the caudal region), *13* port hole, *14* peritoneum, *15* arcuate line, *16* preperitoneal region in the retropubic space

The anterolateral abdominal wall is formed by four paired muscles, which are interconnected in the midline via the linea alba with the muscles of the opposite side and form a functional unit. Coming from lateral/lumbar, the three lateral muscles (transversus abdominis, internal oblique, and external oblique) end at the ipsilateral rectus sheath. The medial end of the muscle fibers of the transversus abdominis constitutes the linea semilunaris (Spieghelian line). Cranially, muscular fibers of the *transversus abdominis* insert in a fan-like fashion at the posterior rectus sheath (Fig. 1/6). The *intercostal nerves* reach the rectus abdominis coming from the lateral side in the layer between the transversus abdominis and the internal oblique (Figs. 1/10 and 2a, b). At the *costal arch margin*, the diaphragm inserts cranially, the transversus abdominis inserts medially (at the sternocostal angle), and the internal oblique inserts caudally. The costal arch margin, which is exposed during the r‑TAR preparation, is the leading structure and is also described as the “watershed” because of the typical distinctiveness of the directions of the muscular insertions (Fig. 1/5). The posterior rectus sheath ends caudally in the region of the *arcuate line* (semicircular line of Douglas; Fig. 1/15). Caudal to the arcuate line, the rectus abdominis is covered exclusively by its fascia (fascia recti propria, which is part of the endoabdominal fascia) and by peritoneum.

**Fig. 2:**
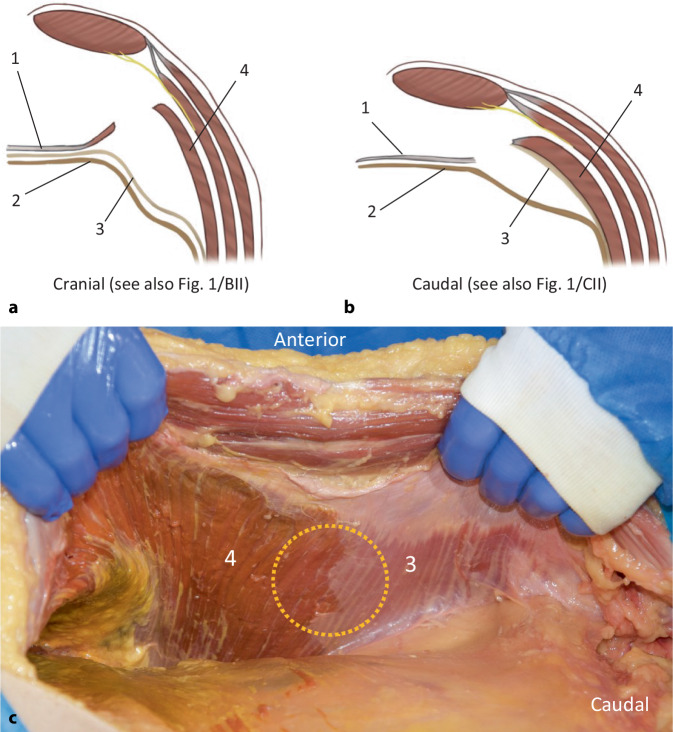
Anatomy of the transversus abdominis release (TAR), left side of the abdominal wall. **a** Transverse section of the preparation plane in the cranial region, where the posterior layer contains the peritoneum and fascia of the transversus abdominis (synonym: endoabdominal fascia). **b** In the caudal region, the fascia of the transversus abdominis remains attached to it, and the posterior layer is formed only by peritoneum and preperitoneal adipose tissue. **c** Illustration of the TAR preparation on a cadaver (figure courtesy of Miguel Garcia-Ureña). *Yellow circle* in the cranial region (*4*), the endoabdominal fascia (including fascia diaphragmatica and fascia of the transversus abdominis) is part of the posterior layer; in the caudal region (*3*), the fascia of the transversus abdominis remains attached to the transversus abdominis. *1* posterior rectus sheath, *2* peritoneum, *3* endoabdominal fascia or fascia of the transversus abdominis, *4* transversus abdominis. (**a,** **c** from [12])

A total of three guiding structures are important for transversus abdominis release:

- Cranially, the insertion of the transversus abdominis on the posterior rectus sheath (Fig. 1/6),
- In the middle section, the lateral border of the posterior rectus sheath, and
- Caudally, the upper end of the retroinguinal space (Bogros’ space) at the arcuate line (Fig. 1/15).

Filip Muysoms rightly remarked that the established TAR terminology is not entirely correct and that it is de facto a “posterior rectus sheath release”.

The *parietal peritoneum* has six layers:

- Mesothelium
- Basal membrane
- Superficial wave-collagen layer
- Superficial elastic network
- Deep elastic network and
- Deep collagen elastic layer

In the region of the posterior abdominal wall and diaphragm, the peritoneum has a rich lymphatic capillary network, with a superficial and a deep layer [6]. The peritoneum has also *lymphatic stomata* (first described in 1863 by Friedrich Daniel von Recklinghausen, who worked in Wuerzburg) connecting the peritoneal cavity with the submesothelial lymphatic capillary network; these peritoneal lymphatic stomata occur predominantly on the peritoneum of the diaphragm, falciform ligament, ovaries, and pelvis [7]. From an ultrastructural point of view, the apical microvilli and the intercellular junctions are of special interest; stomata are often located near the “milky spots” and arise at the junction of three mesothelial cells. Lymphatic stomata are the most important structures for the drainage of peritoneal fluid (up to 100 ml/day under physiological conditions); the negative intrathoracic pressure and diaphragmatic movements influence the intraperitoneal hydrostatic pressure and move the fluid upwards [8]. The mesothelium regenerates very fast, due to metaplasia of subperitoneal fibroblasts [9].

When the posterior rectus sheaths are dissected and detached from the xiphoid, the *fatty triangle* described by Joachim Conze is seen [10]. Correct preparation of the fatty triangle allows the mesh to be positioned posterior to the xiphoid during retrorectal repair (in open procedures as well as in the r‑TAR), ensuring wide overlap of the mesh in the cranial pole of the incisional hernia (Fig. 1/3). Cranial to the fatty triangle is the central tendon of the diaphragm. By hanging from the anterior abdominal wall and underlining the posterior face of the linea alba, the falciform ligament (which is broadly filled with preperitoneal adipose tissue) forms a continuous connecting layer between the right and left posterior rectus sheaths when the posterior rectus sheaths are detached from the xiphoid (Fig. 1/3; [4, 10]). The caudal dissection of the posterior rectus sheath ends at the arcuate line (Fig. 1/15), and here begins the preperitoneal space that ends medially in the *retropubic space* (Fig. 1/16) and laterally toward the iliac fascia in the *retroinguinal space*. The lateral detachment of the peritoneum and the endoabdominal fascia from the transversus abdominis extends dorsally behind the renal fascia (Gerota’s fascia) to the quadratus lumborum.

According to the current International Classification of Abdominal Wall Planes (ICAP), in the r‑TAR procedure the plane “H” (retromuscular plane) is prepared, which is formed anteriorly by the rectus abdominis and the transversus abdominis and posteriorly by the posterior rectus sheath (medial) plus the transversalis fascia (lateral) and, below the arcuate line, by the transversalis fascia only [11]. We miss in the ICAP definition a distinction between the endoabdominal fascia and the portion of the endoabdominal fascia belonging to the transverse abdominis (transverse abdominis fascia), which can lead to terminological confusion especially in distinction to the transversalis fascia, which is weakened in medial inguinal hernias. Here, a discrepancy between the terminology consensus of ICAP and the preparation findings in r‑TAR also seems evident: both on anatomy specimens (e.g., in Fig. 2c) and in r‑TAR operations, the typical finding is that in the cranial portion of the inner abdominal wall the fascia of the transversus abdominis (named transversalis fascia by Parker et al.) is separated from the muscle and firmly attached to the peritoneum, whereas in the caudal region it is firmly attached to the transversus abdominis (Figs. 1/12 and 2/3; [12]). Possibly, the terminological consensus was somewhat premature and the voices of hernia experts without clinical or anatomical experience with the TAR procedure were given weight. Further studies are needed in this regard. In the cranial region, where the fascia of the transverse muscle is broadly detached from the muscle fibers (Figs. 1/9 and 2/4), there may be potential small bleeding sites from the capillary network of the *epimysium* and *perimysium*.

## Robotic transversus abdominis release

The WHO team time-out is mandatory, followed by repetition of the surgical steps on the intraoperative checklist (supplementary material 1). The pneumoperitoneum is created via Veres needle left-subcostal (12 mm Hg). The first three of a total of six ports are initially positioned left-laterally (consider ropivacaine infiltration). We work on the DaVinci Xi with four arms covered. For the left side docking, arms #2 (8 mm), #3 (8 mm, optic), and #4 (12 mm) are used; for the right side docking, arms #1 (8 mm), #2 (8 mm, optic), and #3 (8 mm) are used. The instruments are a 30° optic, the monopolar scissors (Hot Shears MCS), with which we do all the hemostasis, the Prograsp Forceps and the needle holder (Mega SutureCut Needle Driver). Alternatively, a bipolar grasping forceps (Fenestrated Bipolar Forceps or Maryland Bipolar Forceps) can be used to dissect and coagulate. Initially, we work through the ports on the left side of the patient, the DaVinci Xi patient cart is at the feet of the patient, and the boom of the robot is aligned manually (the DaVinci Xi does not have a specific targeting program for access from the feet). The procedure begins with exploratory laparoscopy, and depending on the findings, complete adhesiolysis of the anterior abdominal wall is performed first.

### Step 1 (supplementary material online video 00:40 min).

Start dissection on the right side via the left-sided trocars. Opening of the posterior rectus sheath at the medial margin (the actual hernial margin) from the xiphoid to the retropubic space. The posterior rectus sheath is exposed to its lateral border; care is taken to protect the epigastric vessels and the intercostal nerves, which come from the lateral side and enter into the rectus abdominis muscle.

### Step 2 (supplementary material online video 01:53 min).

**Fig. 3:**
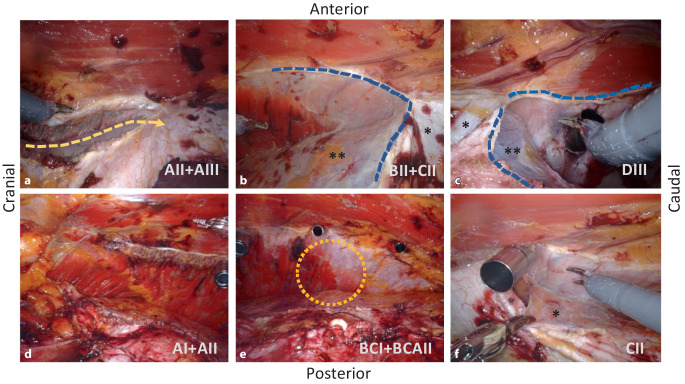
Intraoperative situs of robotic transversus abdominis release (r-TAR), left side of abdominal wall. The abbreviations in the right-bottom sections of the figure correspond to the grids of Fig. 1. **a** Release of the muscular insertion of the transversus abdominis in the area of the posterior rectus sheath (*yellow dashed arrow*). This corresponds to the entry from cranial or “top-down”. **b** Lateral detachment of the posterior rectus sheath (*dashed blue line*) and endoabdominal fascia/peritoneum from the transversus abdominis. **c** Entry to the transversus abdominis release from caudal or “down-to-up” (*dashed blue line*). **d** View after completion of the TAR in the region of the costal margin with the confluence of the diaphragm, the transversus abdominis and the rectus abdominis (see also Fig. 1). **e** View of the completed TAR on the left side; *orange circle* shows the demarcation of the fascia of the transversus abdominis, which remains cranial to the peritoneum and caudal to the muscle. **f** Detachment of the endoabdominal fascia and peritoneum from the transversus abdominis in the area of a port passage; this extraperitonealizes the port. *Posterior rectus sheath, **endoabdominal fascia with peritoneum

The lateral detachment of the posterior rectus sheath can start either cranially (top-down) or caudally (bottom-up). Starting top-down (Fig. 3a), the fibers of the transversus abdominis that insert at the posterior rectus sheath are transected and the endoabdominal fascia (or fascia of the transversus abdominis) is detached laterally; from there, the dissection is continued in both directions, cranially across the costal margin far onto the diaphragm (Fig. 3d), and caudally (Fig. 3b) into the retroinguinal space and the retropubic space. The insertion of the posterior rectus sheath is separated also from the xiphoid, thus, providing wide access to the diaphragm and dissection of the fatty triangle. Care must be taken in this area to avoid damaging muscular fibers of the diaphragm, which can be mistaken for the transversus abdominis at the costal margin (Fig. 3d). In bottom-up fashion, entry to the TAR is started at the arcuate line (Fig. 3c) and continues in cranial direction until the transversus abdominis fibers are transected and the fatty triangle and diaphragm are exposed. Strikingly, the fascia of the transversus abdominis remains attached to the peritoneum in the cranial portion of the dissection (Figs. 1/9 and 2/4) and adheres to the transversus abdominis in the caudal portion (Figs. 1/12, 2/3 and 3e). Laterally, the dissection is continued far lumbar until the detached inner abdominal wall layer lies loosely on the intestinal loops. Using the Prograsp Forceps, this is now pulled medially to check for tension-free conditions for subsequent suture closure. The contralateral ports are inserted under visualization. This concludes the first half of the preparation and the DaVinci Xi is undocked.

### Step 3 (supplementary material online video 03:46 min).

**Fig. 4:**
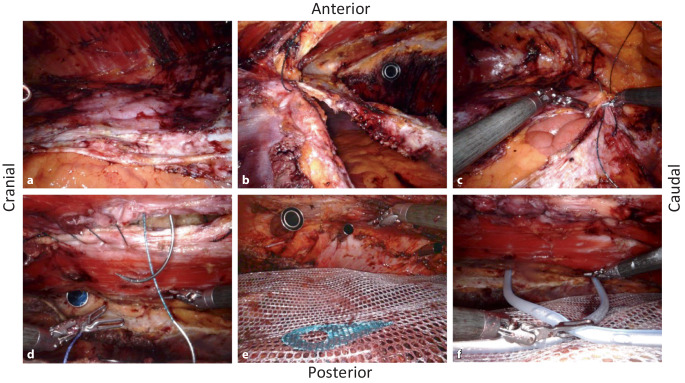
Intraoperative situs of the robotic transversus abdominis release (r-TAR), view from the right side to the left. **a** Situs of the detached “TAR layer”; the layer lies loosely on the bowels; the intestinal contours are clearly visible due to the active peristalsis. **b** Running suture of the posterior TAR layer, starting first from cranial (fatty triangle). **c** Running suture of the posterior TAR layer, starting also from caudal (retropubic space). **d** Running suture of the hernia gap at the Linea alba. **e** Extraperitonealized 30 × 30 cm large mesh in the retrorectus and TAR space. **f** Positioning of 2 silicone drains (one to the diaphragm and one into the retropubic space)

We manually rotate the tripod of the DaVinci Xi arms by 180° (press the side button on arms #1 and #4) and dock three arms manually (without system targeting) to the ports of the patients’ right side. Now the abdominal wall at the left side is prepared in an analogous manner. During the lateral detachment of the posterior rectus sheath and the endoabdominal fascia, the former transabdominally positioned first three ports are now “in the way” and must be extraperitonealized, one after the other, so that the preparation can be continued laterally. The resulting three peritoneal holes (Figs. 1/13 and 3f) are closed with absorbable suture. Finally, the left-sided inner abdominal wall layer is medialized to check that it is free of tension for the subsequent suture closure (Fig. 4a).

### Step 4 (supplementary material online video 05:32 min).

the posterior rectus sheaths, lying tension-free on the bowels, are closed medially with 30 cm long V‑Loc 180/0 USP suture with GS-21 needle (Medtronic, Germany); we start with two sutures, one coming from cranially (from the area of the fatty triangle; Fig. 4b) and one caudally, coming from the retropubic space (Fig. 4c). After the fascia is closed, the pneumoperitoneal pressure is reduced to 4 mm Hg over 3 min to detect any small bleeding from the transversus abdominis and to complete hemostasis. The decision is now made as to whether the anterior rectus sheaths (linea alba) will also be closed robotically, or whether the procedure should require an open skin resection (hybrid version of the procedure).

### Step 5 (supplementary material online video 07:19 min).

With reduced pneumopreperitoneal pressure (8 mm Hg), the median linea alba is gradually closed with V‑Loc 180/0 USP suture (with GS-21 needle), coming from both directions, subxiphoidal and suprapubic (Fig. 4d). The hernial sac is also grasped in the running suture at some points, for seroma prophylaxis. In the hybrid version, instead of step 5, proceed to step 6.

### Step 6 (supplementary material online video 09:29 min).

The Versatex mesh (polyester, monofilament, large pores; Medtronic Germany), with a size of 30 × 30 cm, for example, is inserted via the 12 mm port and spread out so that it underlies the xiphoid in the region of the fatty triangle and extends from there all the way to the symphysis; laterally, the mesh extends on both sides to the lumbar region (Fig. 4e). The mesh does not need to be fixed due to the effectively wide overlap. The perforations of the ports through the muscular abdominal wall are underlaid by the mesh and do not need to be sutured.

### Step 7 (supplementary material online video 10:26 min).

Finally, the large surface of the preparation is sprayed with Arista AH (absorbable hemostyptic; BD Germany) using a FlexTip XL‑R applicator (BD Germany) to ensure capillary hemostasis. Two silicone drains are inserted via the left-sided ports, one to the subdiaphragmatic region (drain #1) and one into the retropubic space (drain #2; Fig. 4f). Counting control of instruments and surgical materials. Relief of the pneumoperitoneum under visualization.

In the hybrid version of the procedure (rh-TAR), mesh positioning, Arista application, and drain placement are performed robotically as described above (steps 6 and 7), and only then is undocking performed and the procedure completed openly. Skin scar and subcutaneous hernia sac are resected, the linea alba is reconstructed with a continuous Everett suture (named after William G. Everett, of Cambridge, England, 1970; with a 4:1 suture length-to-wound length ratio), and the skin is closed with intracutaneous suture.

## Casuistics and study design

This video article summarizes the experience of the operations which were performed from June 2019 to December 2020. It is a cohort study without a control group. The study was approved by the responsible ethics committee of Northwestern Switzerland (Ref. No. 2019-02046). The decision whether surgery was performed exclusively robotically or in a hybrid version was based on the respective findings of individual patients. Patients were followed up clinically 6 weeks postoperatively and by sonography as needed. The 1‑year follow-up is planned in this cohort but was not available at the time of publication. All data were recorded pseudonymously in an in-clinic database that is password-protected and accessible only to the investigators. The *t*-test was used to compare the amount of drainage fluid, length of time the drain was left in place, and inpatient stay. A *p*-value less than 0.05 was considered significant.

## Results

In all, 13 patients were included, mean age was 58.2 years (range 38–74), 30.8% were women, and the mean body mass index (BMI) was 29.9 kg/m^2^ (range 24.8–37.2). The most common secondary diseases were arterial hypertension (76.9%) and chronic obstructive pulmonary disease (COPD; 30.8%). Four patients received oral anticoagulation (30.8%), 8 patients were ASA (American Society of Anesthesiology) category II (61.5%) and 5 patients were ASA category III (38.5%; Table 1).

**Table 1:** Demographic data

	r‑TAR/rh-TAR (*n* = 13)		
		Range	
**Age (mean [SD])**	58.2	38–74	(±12.6)
**Female (*n*** **[%])**	4	–	(30.8)
**BMI, kg/m**^**2**^** (mean [SD])**	29.9	24.8–37.2	(±4.0)
**Smoking (*n*** **[%])**	7	–	(53.8)
**Ethnicity (*n*** **[%])**			
*North-European*	11	–	(84.6)
*Mediterranean*	2	–	(15.4)
**Type of work (*n*** **[%])**			
*Desk-based*	3	–	(23.1)
*Heavy-physical*	2	–	(15.4)
*No labour or retired*	5	–	(38.5)
*Unknown*	3	–	(23.1)
**Comorbidities (*n*** **[%])**			
*Arterial hypertension*	10	–	(76.9)
*Coronary disease*	1	–	(7.6)
*Diabetes*	3	–	(23.1)
*COPD*	4	–	(30.8)
*Previous thrombembolic event*	–	–	–
*Immunosuppressant*	–	–	–
*Oral anticoagulation*	4	–	(30.8)
DOAC	2	–	(15.4)
Marcoumar	–	–	(0.0)
Platelet aggregation inhibitors	2	–	(15.4)
*ASA score*			
I	–	–	–
II	8	–	(61.5)
III	5	–	(38.5)
*CCI (mean [SD])*	9.3	–	(±12.7)

*range* range of variation, *SD* standard deviation, *ASA* American Society of Anesthesiology, *DOAC* dual oral anticoagulation, *CCI* Charlson Comorbidity Index, *COPD* chronic obstructive pulmonary disease, *r‑TAR/rh-TAR* robotic transversus abdominis release/hybrid variation

All hernias were incisional hernias, in one case there was a combination of a median hernia with a paramedian one (after reversal of an ileostomy), and in one patient there was a paramedian incisional hernia (8 × 8 cm, after reversal of a colostomy). Most common causes of incisional hernias were surgery for colorectal cancer (46.1%) and surgery for abdominal aortic aneurysm (15.4%); 3 patients had recurrent incisional hernias (1 after laparoscopic intraperitoneal onlay mesh [IPOM], 2 after open retromuscular mesh repair). The width of the hernia gaps varied from 7–16 cm; in all patients the abdominal wall was morphologically reconstructed. The average ratio of mesh size to hernia gap size was 8.2. Surgical time (incision-to-suture time) averaged 223.5 min (range 167–317 min), including the time spent for docking of the DaVinci Xi, intraoperative redocking, and hybrid skin resection, if applicable. A total of 4 patients (20.7%) underwent the hybrid version of the procedure. All other data on hernia gaps, mesh size, and drains are shown in Table 2.

**Table 2:** Hernia and procedure characteristics

	r‑TAR/rh-TAR (*n* = 13)		
		Range	
*Hernia type (n [%])*			
Umbilical, epigastric or Spieghelian	–	–	–
Incisional	13	–	(100.0)
*Previous disorder or procedure leading to hernia*			
Colorectal cancer	6	–	(46.1)
Abdominal aortic aneurysm	2	–	(15.4)
Recurrence of primary ventral hernia	3	–	(23.0)
Other, benign	3	–	(23.0)
*Size of the hernial gap*			
Length in cm (mean, range [SD])	14.9	8–24	(±4.9)
Width in cm (mean, range [SD])	11.1	7–16	(±3.0)
Defect area in cm^2^ (mean, range [SD])	132.4	88–301	(±69.6)
*Defect closure (n [%])*	13	–	(100.0)
*Size of mesh*			
Length in cm (mean, range [SD])	31.7	29–45	(±4.7)
Width in cm (mean, range [SD])	28.8	25–30	(±2.1)
Area in cm^2^ (mean, range [SD])	907.5	783–1125	(±80.2)
*Mesh area to hernia gap area ratio (mean, range [SD])*	8.2	3.7–15.6	(±3.2)
*Type of mesh (n [%])*			
Versatex	13	–	(100.0)
*Mesh fixation (n [%])*			
None	12	–	(99.3)
Vicryl suture	1	–	(7.6)
*Drain placement (n [%])*	10	–	(76.9)
*Hybrid version (rh-TAR)*	4	–	(20.7)
*Arista application*	8	–	(61.5)
*Skin-to-skin time in min (mean, range [SD])*^a^	223.5	167–317	(±43.5)

*Range* range of variation, *SD* standard deviation, *r‑TAR* robotic transversus abdominis release, *rh-TAR* robotic hybrid transversus abdominis release

^a^Time includes docking, adhesiolysis, and redocking

**Fig. 5:**
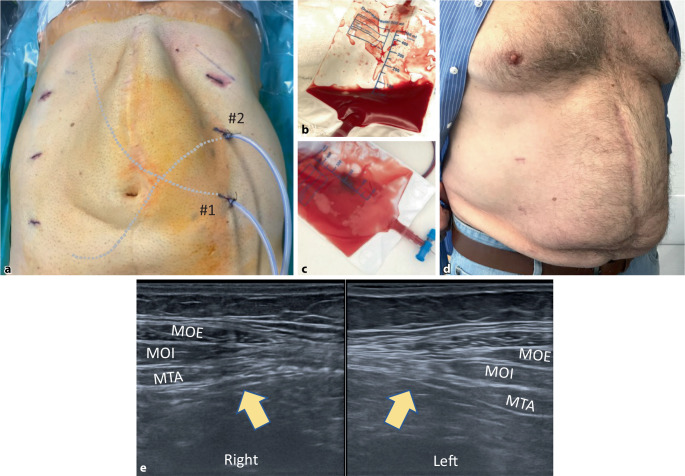
Postoperative course after robotic transversus abdominis release (r-TAR). **a** Final aspect after completion of surgery, showing the 3 accesses on each side as well as the two silicone drains (*#1* is located subphrenically, *#2* is located in the retropubic space); clearly visible is the subcutaneous bulge after endoscopic suture of the linea alba. **b** Bloody drainage secretion on the first postoperative day and **c** clear quality change of the drainage content to serous from the postoperative day 2 onwards. **d** Same patient as after 3 months. **e** Transverse ultrasound image of the linea semilunaris bilaterally (*yellow arrows*); 3 months after r‑TAR, there is neither retraction nor atrophy of the transversus abdominis. *MOE* external oblique; *MOI* internal oblique; *MTA* transversus abdominis

Table 3 shows the postoperative course. The average inpatient stay was 4.7 days. Subphrenic drains drained more than pelvic drains on average (246 ml vs. 145 ml), but without statistical difference (*p* = 0.181; Fig. 5b, c). Wound events occurred in 5 patients: 2 seromas (15.3%) and 3 hematomas (23%; [13]). No wound healing impairment nor wound infection occurred. Patients were mobilized on the evening of the day of the surgery and were given a light dinner. On the first or second postoperative day, all patients had bowel movements. Two patients were revised for postoperative bleeding (Dindo–Clavien IIIb)—the first in open technique, the second using the robotic technique; however, no source of bleeding was found in either patient during hematoma clearance [14].

**Table 3:** Postoperative course

	r‑TAR/rh-TAR (*n* = 13)			
		Range		*p* value
**Hospital stay in days (mean [SD])**	4.7	–	(±2.9)	–
**VAS on POD 1 (mean [SD])**	3.7	–	(±2.4)	–
**Drainage fluid quantity (ml)**				
*Drain #1 (subphrenic) (mean, range [SD])*	246.3	40–560	(±171.5)	*p* = 0.181
*Drain #2 (hypogastrium) (mean, range [SD])*	145.6	20–410	(±150.1)	
**Drains in situ (days)**				
*Drain #1 (subphrenic) (mean, range [SD])*	3.1	1–5	(±1.2)	*p* = 0.292
*Drain #2 (hypogastrium) (mean, range [SD])*	2.5	1–5	(±1.0)	
**Adverse events within 6 weeks**				
*Surgical site occurrence (n [%])*	5	–	(38.4)	–
Seroma (*n* [%])	2	–	(15.3)	–
– Grade I	–	–	–	–
– Grade II	1	–	(7.6)	–
– Grade III	1	–	(7.6)	–
– Grade IV	–	–	–	–
Hematoma (*n* [%])	3	–	(23.0)	–
Surgical site infection (*n* [%])	–	–	–	–
*Unplanned presentation due to pain*	–	–	–	–
*Bowel obstruction (n [%])*	–	–	–	–
*Thrombembolic event (n [%])*	–	–	–	–
*Clavien–Dindo (n [%])*	4	–	(30.7)	–
Grade I	4	–	–	–
Grade II	–	–	–	–
Grade IIIa	1	–	–	–
Grade IIIb	2	–	–	–
Grade IV	–	–	–	–
**Follow-up after 6 weeks (*n*** **[%])**				
*Completed*	13	–	(100.0)	–
*Hernia recurrence*	–	–	(0.0)	–
*Persistent abnormal pain*	–	–	(0.0)	–
*Persistent seroma*	1	–	(7.6)	–
*Persistent hematoma*	1	–	(7.6)	–

*range* range of variation, *SD* standard deviation, *VAS* Visual Analog Scale for Pain Assessment, *POD* postoperative day, *SSO* Surgical Site Occurrence, *r‑TAR* robotic transversus abdominis release, *rh-TAR* robotic hybrid transversus abdominis release

## Discussion

The development of the modern-day TAR began in 2008, when Alfredo Carbonell described posterior component separation (pCS) for open incisional hernia repair [15]. In pCS, the plane between the internal oblique and the transversus abdominis is dissected laterally of the posterior rectus sheath (plane “E” or retro-oblique plane in ICAP; [11]). However, there is a significant risk of damage to intercostal nerves, which run exactly in this plane. Already 4 years later, Yuri Nowitzky described the open TAR, a technical modification of the pCS which spares the neurovascular bundles and corresponds to the plane “H” or the retromuscular plane in ICAP [11, 16]. In a study on cadavers, the medialization effect of the various component separations that have been published were investigated; the pCS allows a medialization of at mean 9.4 cm per side, which is more than the 5.8 cm per side reached on average by the anterior component separation (described by Alfonso Albanese in 1951 and by Osacar Ramirez in 1990; [17]). It can be assumed from anatomical considerations that the TAR (as a variant of pCS) allows at least a comparable medialization as the pCS.

In recent years the laparoscopic IPOM (intraperitoneal onlay mesh) technique has become very common and is mainly known for its low wound complication rate. A recent meta-analysis comparing the different approaches shows that meshes in retrorectal position have the lowest recurrence rate (odds ratio [OR] 0.281, 95% confidence interval [CI] 0.06–0.47) with also low risk of mesh infections (OR 0.449, 95% CI 0.12–1.16), and IPOM having a lower risk of wound complications (OR 0.878, 95% CI 0.29–1.99); this meta-analysis concludes with 94.2% probability, that “retromuscular” is the best layer for mesh implantation [18]. Other arguments against the laparoscopic IPOM are an increased recurrence rate, increased postoperative pain, and long-term complications on the mesh (adhesions and mesh erosions), so that there is a tendency among surgeons to extraperitonealize the mesh by minimally invasive approaches.

The r‑TAR was first described in 2017 by Warren et al. [19]. In the original description, the robot is docked from both sides and the lateral dissection extends to the projection of the anterior axillary line; that series compared the laparoscopic approach (*n* = 103) with robotics (*n* = 53) including different techniques, whereby 43% of robotic procedures where performed as r‑TAR [19]. In a retrospective study comparing open TAR (o-TAR; *n* = 76) and r‑TAR (*n* = 26), the operating room time was significantly shorter with o‑TAR (287 vs. 365 min), but the morbidity was higher with o‑TAR (39 vs. 19%, *p* = 0.09) and the hospital length of stay was significantly longer with o‑TAR (6 vs. 3 days; [20]).

A further development of r‑TAR is the hybrid version of the procedure (rh-TAR), in which the skin scar and hernia sac are resected at the end of the procedure and the linea alba is closed by open technique. In a cohort study of 20 rh-TAR surgeries, Kudsi et al. [21] showed that the complication rate was low and patient satisfaction was high, as measured by the European Hernia Society (EHS) quality of life (QoL) score (cosmetic satisfaction and disability at work where significant). The operative time of rh-TAR in the series of Kudsi was 296.5 ± 94.5 min, the hernia gap area varied from 204–333 cm^2^, the mesh area from 600–1050 cm^2^, the ratio of mesh area to hernia gap area was 4.11 on average; there were 3 seromas (15%) and 2 wound infections (10%), 2 patients required wound revision (10%), and no recurrence occurred with an average follow-up of 319 days [21]. In the literature, r‑TAR is recommended for hernia gaps with a width of 7–14 cm, while for gaps below that (4–7 cm) r‑Rives (retrorectal mesh) is a good option for incisional hernias [3, 22].

In our series, the operation time was 217 min on average (range 167–317 min), which was slightly less than the average in the literature; however, the hernia gap area was also slightly smaller at 132.4 cm^2^ (range 88–301 cm^2^) and the BMI was lower at 29.9 kg/m^2^ (range 24.8–37.2 kg/m^2^) than, for example, that reported by Kudsi et al.(hernia gap area 255 cm^2^ and BMI 33.5 kg/m^2^) [21]. In the initial phase, we performed two revisions due to hematoma. As a consequence, we adapted our surgical protocol (see also supplemental material 1) with three measures:

- Inspection of hemostasis after suturing the posterior rectus sheaths under reduced pneumoperitoneum (4 mm Hg over 3 min),
- Application of Arista on the mesh to support capillary hemostasis on the large component separation surface area, and
- Insertion of 2 silicone drains (Robinson drains, without suction), one subphrenic and one retropubic.

After this adjustment to the technique, no patient revisions were necessary. It seems that, judging by the quality and quantity of the drainage secretion, the extensive detachment of the peritoneum from the diaphragm and the entire abdominal wall leads to a temporary disruption of the physiological resorption mechanisms of the peritoneal fluid. One explanation may be the disruption of the lymphatic pathways and the peritoneal stomata, especially in topography of the diaphragm. Further studies are needed in this regard. A major advantage of r‑TAR is that the bowels are hardly touched (no-touch of the intestinal serosa during the entire procedure), which is demonstrated postoperatively by the low incidence of ileus and the possibility of immediate food build-up. Tears of the peritoneum occur very rarely and can be sutured without problems.

It is surprising to see that the wide hernia gaps (in our series of up to 16 cm in diameter) are adaptable with suture after 2–3 h under 12 mm Hg pneumoperitoneum, both exclusively robotically (r-TAR) and hybrid robotically (rh-TAR); this is probably an effect of the elongation of the abdominal wall due to muscle relaxation in combination with the simultaneous pneumoperitoneum, an effect that seems to be similar to the open AWEX (abdominal wall expanding) system for closure of the abdominal wall after laparostomy [23]. Future studies have yet to further define the effect of abdominal wall distension under pneumoperitoneum during laparoscopic surgery, in order to optimize surgical planning and patient counseling; the goal would be, for example, to extrapolate the expected abdominal wall extension by analyzing the muscle morphology and the hernia gap characteristics on computer tomography (CT) scan images.

Even though esthetic satisfaction of patients after r‑TAR is achieved after 3–6 months with the smoothing of the shape of the abdominal wall (Fig. 5a, d), we have seen, as Yusef Kudsi did, that patient satisfaction is high immediately after rh-TAR surgery [21]. On postoperative ultrasound checks at 3 and 6 months, the transversus abdominis at the level of the linea semilunaris is inconspicuous, with no signs of lateral retraction or atrophy; the morphology and probably also the biomechanics of the abdominal wall are restored after r‑TAR (Fig. 5e).

The issue of optimal mesh size needs to be further evaluated. Current “rules of overlap” fall short of accounting for the high variability of situations they claim to apply to. There is a natural limit for mesh size; this limit is the size of the human anterolateral abdominal wall (including part of the diaphragm, lumbar region, and retropubic space). It is certain that the requirement of a mesh-area-to-hernia-area ratio of 16 applies only to bridging procedures and does not apply to cases where the hernia gap was closed, as is the case with r‑TAR and other techniques of morphologic and functional abdominal wall reconstruction [24, 25]. Mesh fixation at numerous points, as required for bridging procedures, also does not appear to be applicable to large-area extraperitoneal meshes in combination with hernia gap closure [26]. Botulinum toxin-assisted chemical component separation is probably not necessary for most of the r‑TAR procedures. The fact that the cost of r‑TAR is significantly lower than that of laparoscopic IPOM is mentioned only in passing [3].

The r‑TAR is not an intervention for beginners in robotics. We recommend that those interested in the method repeat the anatomy on the cadaver and gain experience with the robotic Rives at the beginning [3]. It is also useful to perform the first procedure with the assistance of a proctor who is familiar with the method. Especially the risk of diaphragmatic lesion, the complexity of the preparation of the fatty triangle, the protection of the intercostal nerves and the navigational safety in the retroinguinal space are demanding. Challenging may be also the suture of the linea alba. The r‑TAR is (borrowing a phrase from Patrick Süsskind’s *Perfume: The Story of a Murderer,* 1985) the most ingenious and innovative implementation of everything we have learned about abdominal wall reconstruction in the last three decades, three decades exceedingly rich in ingenious and innovative ideas about abdominal wall reconstruction. The r‑TAR is emerging as the supreme discipline in hernia repair, and minimally invasive extraperitonealization of large meshes is probably its greatest contribution. Further studies are needed to confirm or refute the present results.

## Key points for practice

- The robotic transversus abdominis release (r-TAR) procedure combines the advantages of open repair (morphological and functional reconstruction) with the advantages of laparoscopy (few wound complications, low postoperative ileus, short hospital stay).
- Hernia gaps of 8–14 cm in width can be closed.
- r‑TAR allows extraperitonealization of large meshes.
- Advanced anatomical knowledge is required.
- Monitoring of hemostasis under low pressure is important; postoperative drainage of serous fluid via drains is useful.
- The hybrid version (rh-TAR) results in significant esthetic satisfaction.

## Supplementary Information

Video: Robotic transversus abdominus release

Supplemental material 1: intraoperative checklist

## Declarations

For this work, no studies were performed on humans or animals by the authors. The data collection of this study is based on a positive ethics vote of the Ethics Committee Northwestern Switzerland (No. 2019-02046), and the studies were performed based on the ethical guidelines specified therein. For images or other information in the manuscript that can be used to identify patients, written consent was obtained from the patients and/or their legal representatives.
