# Erratum to: Robotic hernia repair III. English version: Robotic incisional hernia repair with transversus abdominis release (r-TAR). Video report and results of a cohort study

**DOI:** 10.1007/s00104-021-01564-w

**Published:** 2021-12-20

**Authors:** Ulrich A. Dietz, O. Yusef Kudsi, Miguel Garcia-Ureña, Johannes Baur, Michaela Ramser, Sladjana Maksimovic, Nicola Keller, Jörg Dörfer, Lukas Eisner, Armin Wiegering

**Affiliations:** 1 Department of Visceral, Vascular and Thoracic Surgery, Cantonal Hospital Olten (soH), Baslerstrasse 150, 4600 Olten, Switzerland; 2 Department of Surgery, Good Samaritan Medical Center, 235 North Pearl St., 02301 Brockton, MA USA; 3 Hospital Universitario del Henares, Universidade Francisco de Vitoria, 28223 Pozuelo de Alarcón, Madrid Spain; 4 Department of General, Visceral and Vascular Surgery, Cantonal Hospital Baden, Im Engel 1, 5404 Baden, Switzerland; 5 Department of General, Visceral, Transplant, Vascular and Pediatric Surgery, University Hospital Wuerzburg, Oberduerrbacher Strasse 6, 97080 Wuerzburg, Germany

**Erratum to:**

**Chirurg 2021**

10.1007/s00104-021-01500-y

The article “Robotic hernia repair.r-TAR” is part III and a translation of the originally in *Der Chirurg* 10/21 published German language article. The title and subtitle have been amended accordingly. The original article has been corrected.

