# Supplementary material for: Robotic hernia repair III. English version: Robotic incisional hernia repair with transversus abdominis release (r‑TAR). Video report and results of a cohort study
Source: Chirurg. 2021 Sep 8;92(Suppl 1):28–39. doi: 10.1007/s00104-021-01500-y (PMC8695562; doi:10.1007/s00104-021-01500-y)
Supplement: Supplementary file 2 — Supplemental material 1: intraoperative checklist [file 104_2021_1500_MOESM2_ESM.pdf]

Patient-ID

Date: _____/_____/_____

Surgeon: _____________

r-TAR
(rh-TAR)

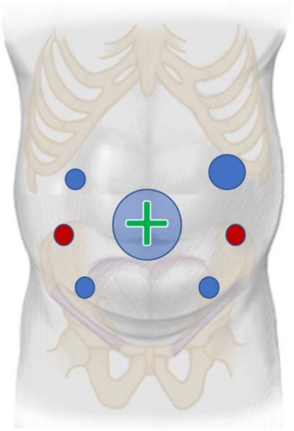

Supien position
Arm positioning not relevant

Intraoperative
Re-dock

Manual targeting

daVinci Anatomy:
- (none)

daVinci Position:
- (none)

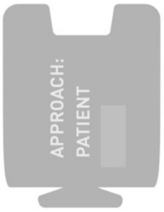

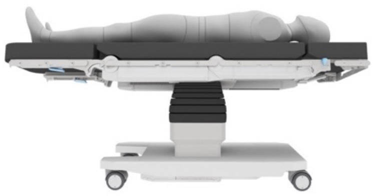

Perioperative Procedures/daVinci Checklist (Note: Does not replace the WHO Team Time-Out).

I have reviewed the patient informed conset sheet and checked it for content and comments: □ Yes □ No
Perioperative antibiotics: □ Yes □ Cefuroxim 1,5g to 3g (Alternative: □ Clindamycin 600mg)
Hybrid-Procedure: □ Yes □ No If yes, inform nurse to prepare additional instruments.

Before docking: **Prepare for double docking.**

Distance of trocar line to the hernia gap is approximately 15cm:	□ Yes	□ No
It is a recurrent hernia:	□ Yes	□ No
Number of previous repairs and history of adhesiolysis known:	□ Yes	□ No
Ropivacaine infiltration of port incisions:	□ Yes	□ No
Laparoscopic visual inspection of port placement	□ Yes	□ No

Console:

Start through left-sided trocars:	□ Yes	□ No
Complete adhesiolysis of the abdominal wall needed:	□ Yes	□ No
Entry to the contralateral rectus sheath at the hernia gap border:	□ Yes	□ No
Transversus abdominis release, top-down (yellow arrow) or down-to-up (green arrow)		
Preparation at costal margin, diaphragm and xiphoid, complete cranial transection of the posterior rectus sheath:	□ Yes	□ No
Preparation in the spaces of Retzius and Bogros:	□ Yes	□ No
Re-dock, analogous preparation from the right, extraperitonealisation of ports	□ Yes	□ No
Check for peritoneal tears/lesions (suture if necessary):	□ Yes	□ No
Median running suture of posterior rectus sheath (V-Loc 0):	□ Yes	□ No
Median running suture of anterior rectus sheath (including hernia sac)(V-Loc 0):	□ Yes	□ No
Measuring of mesh size, roll the mesh for insertion and positioning:	□ Yes	□ No
Revision of hemostasis and application of Arista:	□ Yes	□ No
**Reduction of pneumopreperitoneum to 4mmHg for 3min or RR>160mmHg**	□ Yes	□ No
Positioning of 2 silicone drains (retroxiophoideal and suprapubic):	□ Yes	□ No

Ending:

**Hybrid skin resection:**	□ Yes	□ No
OR-data recorded in the hernia registry:	□ Yes	□ No
Laparoscopic visual inspection of port removal:	□ Yes	□ No

Pre-stationary preparation:
CT abdomen with transverse and
and sagittal sections (morphology and
probability of adhesions)
Dietz incisional hernia classification (2007)

Sutures and materials:
V-Loc 180 (green) 0 USP V-21 (30cm)
Vicryl 3-0 SH (20cm)
Vicryl 3-0 (Sutupak) for mesh rolling up
Ruler
Versatex mesh 30x30cm
Marking pen
2 silicone drains #18
Arista AH
Arista FlexTip XL-R Applicator

Positioning of the patient on OR-table
Supine position
No pink pad needed
Both arms extended

Planned OP time: 4-6h

CIRS record: □ Yes □ No

Instruments:
30° optics
Prograsp Forceps
Mega SutureCut Needle Driver
Hot Shears MCS
5x 8mm daVinci Port
1x 12mm daVinci Port

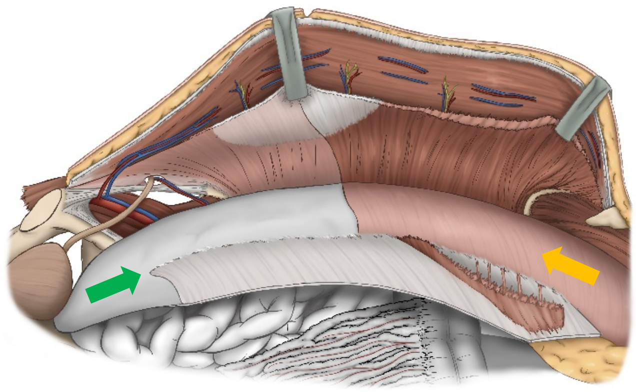

Dietz (Hrsg.) (2021) Offene Hernienchirurgie. Springer Verlag.

Skin closure:
Ropivacaine 0,5% 10ml
Caprosyn 4-0 USP with P-24 needle
Cyanoacrylate-Glue

Postoperative prescription:
PONV-prophylaxis
Mobilisation on the same evening
Small dinner
Thromboembolism prophylaxis
Keep drains for 2-3 days

Swiss-DRG G09D, Target dwell time: **0** | 3 | 9 days

Planned discharge: _____/_____/_____

Dietz at al (2007) J Plast Reconstr Aesthet Surg 60: 383-388.

Ocurrence	M = morphology	S = size (cm)	RF = risk factors (+ → +++)
p (*primary*)<br>r (*recurrent*)	m (*median*)<br>u (*umbilical*)<br>sp (*suprapubic*)<br>sc (*subcostal*)<br>t (*transversal*)<br>l (*lumbal*)<br>pm (*paramedian*)<br>nc (*other*)	a x b	Obesity (BMI >25)<br>Male gender<br>Nicotine abuse<br>Wound contamination<br>Age > 45 years<br>Disease<br>2nd laparot. < 1 month<br>Emergency procedure<br>Postop. Complications<br>…

v/i M… S…x… RF…

Debriefing: What can be done better in the next surgery?
